# Spatial Distributions of GABA Receptors and Local Inhibition of Ca^2+^ Transients Studied with GABA Uncaging in the Dendrites of CA1 Pyramidal Neurons

**DOI:** 10.1371/journal.pone.0022652

**Published:** 2011-07-25

**Authors:** Yuya Kanemoto, Masanori Matsuzaki, Susumu Morita, Tatsuya Hayama, Jun Noguchi, Naoko Senda, Atsuya Momotake, Tatsuo Arai, Haruo Kasai

**Affiliations:** 1 Laboratory of Structural Physiology, Center for Disease Biology and Integrative Medicine, Graduate School of Medicine, The University of Tokyo, Tokyo, Japan; 2 CREST, Japan Science and Technology Agency, Saitama, Japan; 3 Graduate School of Pure and Applied Sciences, University of Tsukuba, Tsukuba, Japan; The Research Center of Neurobiology-Neurophysiology of Marseille, France

## Abstract

GABA(γ-amino-butylic acid)-mediated inhibition in the dendrites of CA1 pyramidal neurons was characterized by two-photon uncaging of a caged-GABA compound, BCMACM-GABA, and one-photon uncaging of RuBi-GABA in rat hippocampal slice preparations. Although we found that GABA_A_-mediated currents were diffusely distributed along the dendrites, currents elicited at the branch points of the apical dendritic trunk were approximately two times larger than those elsewhere in the dendrite. We examined the inhibitory action of the GABA-induced currents on Ca^2+^ transients evoked with a single back-propagating action potential (bAP) in oblique dendrites. We found that GABA uncaging selectively inhibited the Ca^2+^ transients in the region adjacent (<20 µm) to the uncaging site, and that GABA uncaging was effective only within a short period after bAP (<20 ms). The strength of inhibition was linearly related to the amplitudes of the GABA currents, suggesting that the currents inhibited a sustained, subthreshold after-depolarization without preventing propagation of bAP. GABA uncaging at the dendritic branch points inhibited Ca^2+^ transients farther into dendritic branches (>20 µm). Our data indicate that GABA inhibition results in spatially confined inhibition of Ca^2+^ transients shortly after bAP, and suggest that this effect is particularly potent at the dendritic branch points where GABA receptors cluster.

## Introduction

The integration of excitatory and inhibitory signals, played out over space and time, is a key feature of neuronal dendrites in the mammalian central nervous system. This dendritic integration has been investigated most powerfully at the synaptic level using two-photon (2P) uncaging of neurotransmitters. Unlike electrical stimulation, 2P uncaging can stimulate postsynaptic receptors at specific locations within the dendrite. In fact, 2P uncaging of glutamate has elucidated the mechanisms of many key aspects of neuronal function, including the structure-function relationship of dendritic spines [1], the confinement and diffusion of Ca^2+^ signals within dendrites [2], [3], synaptic plasticity [4] and dendritic spike generation [5], [6]. Although the regulation of Ca^2+^ signals by inhibitory input has been investigated with electrical stimulation [7], [8], [9], it has not been characterized with 2P uncaging of the inhibitory neurotransmitter GABA.

Recently, a series of new caged-GABA compounds has been synthesized [10], [11], [12], [13]. Here, we describe the use of these reagents with both one photon (1P) and 2P excitation to examine the distribution and function of GABA receptors in the dendrites of CA1 pyramidal neurons. We observed that functional GABA_A_ receptors were diffusely distributed over most of the dendritic surface, but apparently clustered at the branch points of the apical dendritic trunk. Furthermore, we found that GABA inhibition of bAP-induced Ca^2+^ transients was very confined and particularly potent at these dendritic branch points.

## Results

### Spatial distributions of functional GABA_A_ receptors in the dendrites

**Figure 1:**
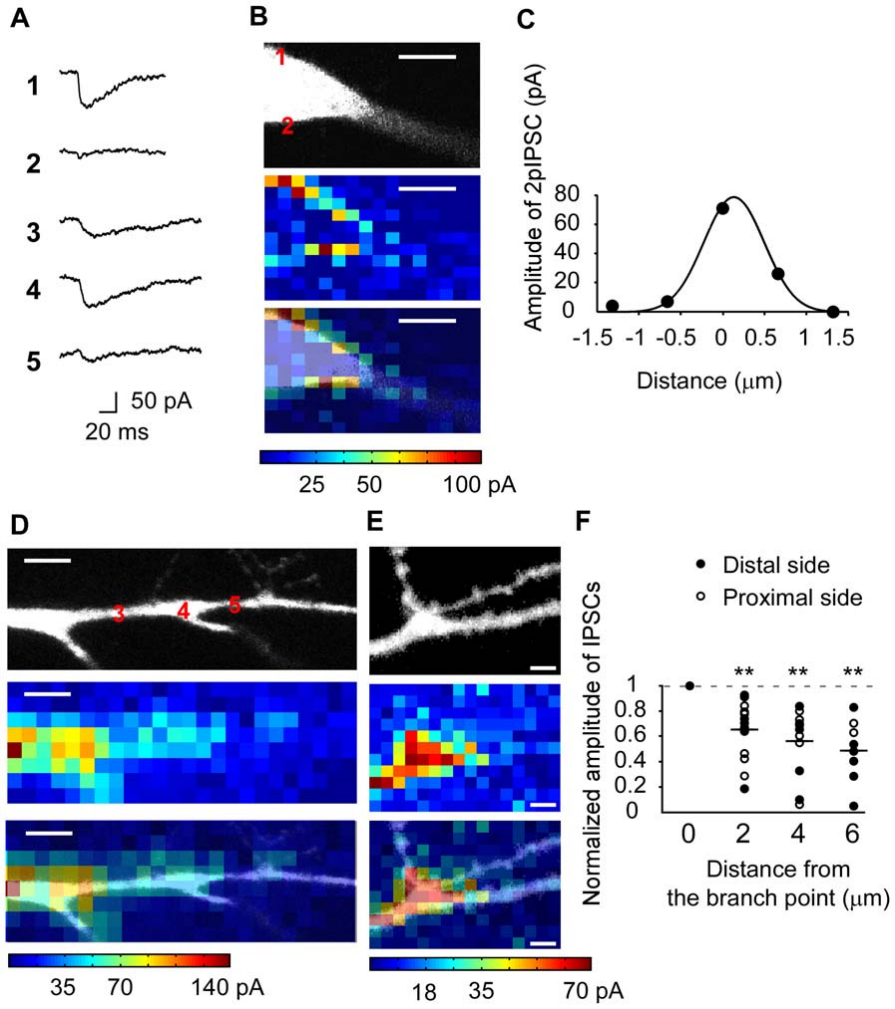
Distributions of GABA receptors in CA1 pyramidal neurons investigated by 2P uncaging of GABA. **A**, Representative traces for IPSCs evoked by 2P uncaging of BCMACM-GABA. Each current trace was evoked at the corresponding numbers on the fluorescence images in (B, D). **B**, **D**, **E**, Mapping of functional GABA receptors. The upper, middle and lower panels represent the fluorescence images, maps of GABA-induced currents, and their overlays, respectively. Scale bar represents 5 µm. (**B**) Two-dimensional (2D) map at the soma. BCMACM-GABA (6 mM) was uncaged with the mode-locked laser at 800 nm (8 mW, 1 ms). The interval between pixels was 0.9 µm. (**D**, **E**) Three-dimensional (3D) maps of apical dendrites. The interval between pixels was 1.6 µm (D) or 1.3 µm (E). We obtained 2D maps at three consecutive z-axis planes with an interval of 5 µm (D) or 1.5 µm (E), and represented with the maximal intensity projection in the 3D maps. **C**, Spatial resolution of 2P mapping. The smooth line represents Gaussian fitting of the data from a GABA hot spot in (B). The FWHM lateral resolution was determined to be 0.85 µm. **F**, Clustering of GABA receptors at the branch points of the major dendritic trunks. Amplitudes of IPSCs along the dendrites were normalized by the maximal amplitude of IPSCs at the branch points. Horizontal bars represent the mean values. Filled and open circles represent data obtained from dendritic locations distal and proximal to the branch point, respectively. The normalized values differ from 1.0 with ** *P*<0.01 (*t*-test, *n* = 8–16).

The distribution of GABA receptors in the dendrites was examined first with 2P uncaging (800 nm) of GABA in whole-cell-recorded hippocampal CA1 pyramidal cells, using a CsCl based intracellular solution (Materials and Methods). Two-photon uncaging was performed with a laser power of 8–10 mW for 1–2 ms, as in the case with caged-glutamate [1]. We used BCMACM-GABA (6 mM) as a caged-GABA compound [10], because it could stably elicit GABA_A_ mediated currents (2pIPSCs)(Fig. 1A) with a coefficient of variation (CV) as small as 0.12. No current was evoked in the absence of the caged-GABA compound. In addition, BCMACM-GABA yielded 2pIPSCs with a more rapid onset and decay (Fig. 1A) than was observed with DCAC-GABA [12], despite their similar caging group. This difference is likely due to a faster uncaging reaction with BCMACM-GABA. Two dimensional (2D) mapping of 2pIPSCs resolved several hot spots around the soma (Fig. 1B). The hot spots were observed only on the periphery of the cell body because of the high Z-axis resolution of 2P uncaging [1].The lateral full-width-at-half-maximum (FWHM) spatial resolution of the mapping was estimated as ∼0.9 µm from the hot spot (Fig. 1C). We used the first to third branches of the apical dendritic trunk in the following experiments.

Three dimensional (3D) mapping of 2pIPSCs along the major trunk of the dendrites revealed that the amplitudes of 2pIPSCs were approximately two times larger at the branching points than in other dendritic regions (Fig. 1D, E). When the amplitudes of 2pIPSCs were normalized by those at branch points, they decayed to 0.59±0.24 (mean ± SD, *n* = 39) within 5 µm (Fig. 1F). The larger amplitudes of GABA-induced currents were not simply explained by the greater membrane area which might be involved in the branch point, because the larger currents were detected even at the smooth part of the dendritic trunk which was a few microns distant from the branch point (Fig. 1D, E), and because our mapping system had a spatial resolution less than 1 µm (Fig. 1C). These findings suggest that the density of GABA_A_ receptors is significantly higher within the dendritic region adjacent to a branching point. We have never found hot spots of GABA-induced currents at the dendritic spines (Fig. 1E), unlike glutamate-induced currents [1].

**Figure 2:**
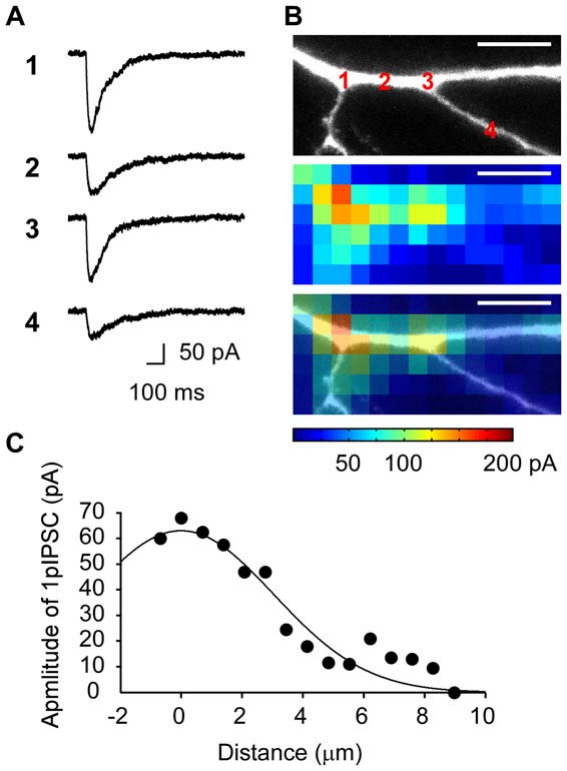
Distributions of GABA receptors investigated with 1P uncaging of GABA. **A**, Representative 1pIPSCs. Numbers correspond to those in the fluorescence image (B). **B**, Two-dimensional map of functional GABA receptors at the apical dendrite. Upper, middle and lower images represent the fluorescence image, the map, and their overlay, respectively. Scale bar represents 10 µm. RuBi-GABA (0.2 mM) was uncaged by the 473 nm laser (0.5 mW, 4 ms) at each pixel. The interval of pixels was 2.7 µm. **C**, Spatial resolution of 1P mapping. The smooth line represents Gaussian fitting of the data shown in (B). The lateral FWHM resolution was estimated as 7.2 µm.

The same tendency was found with 1P excitation (473 nm) of the caged-GABA compound RuBi-GABA (0.2 mM) [11]. The time courses of 1pIPSCs tended to be slower than those of 2pIPSCs (Fig. 2A), likely because GABA was released diffusely as a result of the cone-shaped profile of 1P excitation along the z-axis. We performed 2D mapping of 1pIPSCs in the dendrites (Fig. 2B) using a low power setting (0.5–0.8 mW at specimen for 2–4 ms) to avoid possible damage to the neuron by repetitive laser irradiation. No current was evoked by the laser irradiation in the absence of the caged-GABA compound. We found that 1pIPSCs could be evoked at any point along the major dendritic trunk and oblique dendrites (Fig. 2B) [11]. The lateral FWHM resolution of the mapping was ∼7 µm (Fig. 2C). Consistent with 3D maps of 2pIPSCs, the amplitudes of 1pIPSCs were approximately two times larger at branching points than in other regions (Fig. 2B)—current amplitudes were 0.58±0.17 (mean ± SD, *n* = 4) times smaller in the latter. In our experimental conditions, slow GABA_B_ mediated K^+^ currents were not recorded [7]. We used 1P uncaging in the following study to minimize the concentrations of caged GABA compounds, which may have antagonistic effects on GABA receptors [11], [13].

### Inhibition of spike-induced Ca^2+^ transients by one-photon GABA uncaging

**Figure 3:**
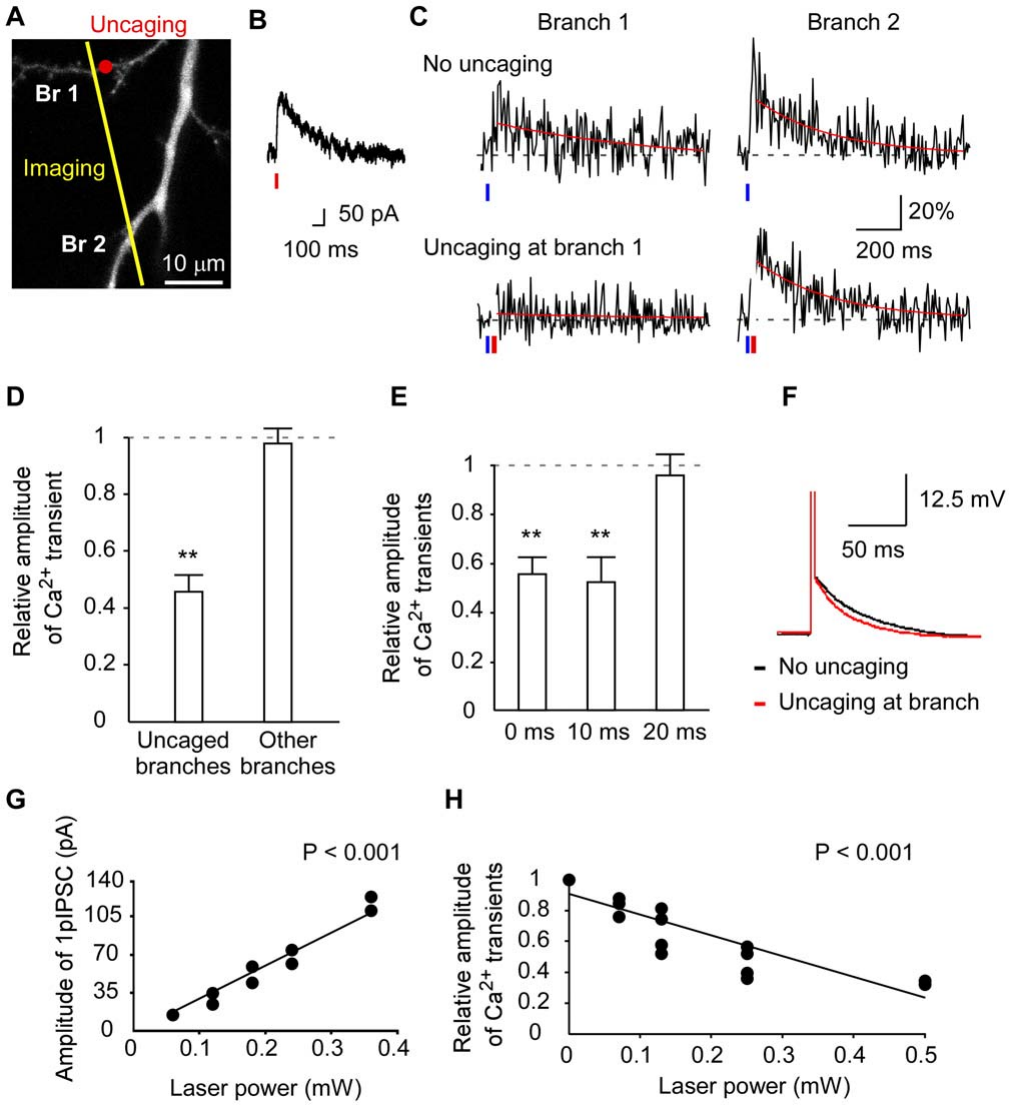
Inhibition of bAP-induced Ca^2+^ transients by uncaging of RuBi-GABA at oblique dendrites. **A**, Fluorescence image (Alexa-594) of a dendrite investigated in (B,C). The red circle in the branch (Br) 1 represents uncaging point. Line scanning was performed along the yellow line for Ca^2+^ measurements. **B**, Current trace of 1pIPSC evoked at −20 mV. Uncaging of RuBi-GABA was applied to the red circle in (A). The red vertical line represents the time of GABA uncaging (0.5 mW, 4 ms). **C**, Normalized changes (Δ*F*/*F*) in fluorescence of OGB-1 (0.1 mM) evoked by a single bAP. Inhibition of the Ca^2+^ transients occurred at branch 1, but not at branch 2. Uncaging was applied 10 ms after bAP. Blue and red vertical lines denote the times for bAP induction and GABA uncaging, respectively. We masked the traces during 473 nm laser irradiation for GABA uncaging. **D**, Inhibition of bAP-induced Ca^2+^ transients in the branches which were applied with GABA uncaging or not. The amplitudes of the Ca^2+^ transients with GABA uncaging were normalized by those without. Bars represent the mean ± SEM. The relative values are different from 1.0 with ** *P*<0.01 (*t*-test, *n* = 8). **E**, Time dependence of Ca^2+^ inhibition by GABA uncaging. Uncaging was applied 0 ms, 10 ms, or 20 ms after bAP induction. Ca^2+^ imaging was performed at the site of uncaging. The relative values differ from 1.0 with ** *P*<0.01 (*t*-test, *n* = 6–10). **F**, Example of the effect of GABA uncaging on spike after-depolarization. **G**, Laser-power dependence of the amplitudes of 1pIPSCs induced by GABA uncaging at the soma. The correlation is significant (*P*<0.001). **H**, Laser-power dependence of inhibition of the Ca^2+^ transients by GABA uncaging at oblique dendrites. The correlation is significant (*P*<0.001).

We imaged Ca^2+^ transients in dendritic branches (Fig. 3A, yellow line) by evoking single back-propagating action potentials (bAP) with current injection to investigate the effect of GABA uncaging at an oblique dendritic branch (Fig. 3A, red circle). The laser power for GABA uncaging was set so that the amplitudes of 1pIPSCs were about 100 pA at the holding potential of −20 mV (Fig. 3B) with the K-gluconate based intracellular solution (Materials and Methods), which corresponded to several (2–5) synchronous spontaneous IPSCs (20–50 pA). The amplitudes of 1pISPCs were far smaller than those used in the experiments with electrical stimulation [7]. The K-gluconate solution was used to study the spread of GABA inhibition in physiological condition.We found that such GABA uncaging selectively reduced the Ca^2+^ transients at uncaged branches while other branches were unaffected (Fig. 3C, D).

GABA uncaging reduced the Ca^2+^ transients only when it was applied within a period shortly after depolarization (Fig. 3E). Thus, the inhibition was detected when GABA uncaging was applied at 0 ms and 10 ms after bAP, but not at 20 ms. Importantly, bAP was followed by after-depolarization at the soma for 50 ms (Fig. 3F), and the depolarization was often diminished by GABA uncaging (8 out of 10 dendrites)(Fig. 3F). It is therefore likely that 1pIPSP inhibited the after-depolarization of bAP at the branches, thereby suppressing the Ca^2+^ transient, and that the inhibition was mediated by GABA_A_ receptors, as it was rapid.The inhibition did not effectively spread into other branches, as analyzed in more detail below.

**Figure 4:**
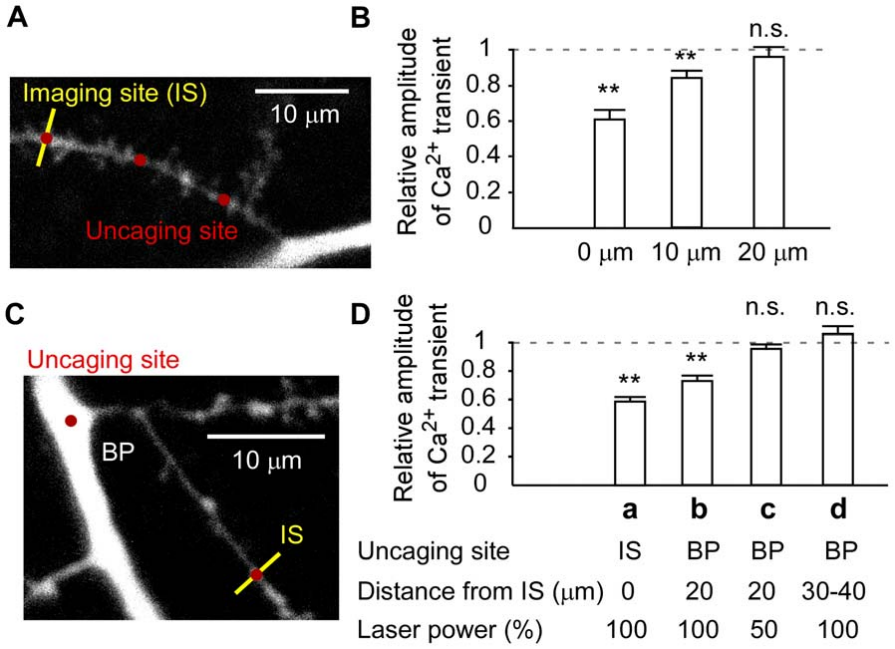
Spatial confinement of GABA inhibition on the Ca^2+^ transients evoked by single bAP. **A**, Fluorescence image (Alexa-594) of the cell where imaging (yellow line) and uncaging (red pints) were applied to an oblique branch. **B**, Distance dependence of inhibition of the Ca^2+^ transients by GABA uncaging. Uncaging of RuBi-GABA was applied at 0 µm, 10 µm, and 20 µm proximal to the imaging site at 0 ms after bAP induction. Bars represent mean ± SEM. The relative values differ from 1.0 with ** *P*<0.01 and n.s. *P*>0.05 (*t*-test, *n* = 5–7). **C**, Fluorescence image of the cell where imaging (yellow line) was performed at an oblique branch, while uncaging (red pints) were applied either at the imaging site (IS) or the branch point (BP) of the major apical dendritic trunk. **D**, Inhibition of the Ca^2+^ transients by GABA uncaging at imaging site (IS) or branch point (BP). Uncaging was applied at IS (a) or BPs which were 20 µm (b, c) or 30–40 µm (d) proximal to the imaging site. Inhibition was applied at 0 ms after AP induction. Laser powers were reduced to 50% in (c) relative to (b), which resulted in significant reduction of inhibition (*P*<0.05 with paired *t*-test, *n* = 6). The relative values differ from 1.0 with ** *P*<0.01 and n.s. *P*>0.05 (*t*-test, *n* = 6–17).

The inhibition of Ca^2+^ transients was graded, based on the observation that the amplitude of 1pIPSCs at −20 mV was proportional to the laser power (Fig. 3G) and because the inhibition of Ca^2+^ transients depended on the laser power in a graded fashion (Fig. 3H). Moreover, the inhibition was local, because it was induced when GABA uncaging was applied at the site of imaging or 10 µm apart, but not when uncaging was applied 20 µm proximal to the imaging site on the same oblique dendrites (Fig. 4A,B). These data indicate that GABA uncaging did not suppress propagation of bAP into dendrites, but rather locally inhibited the after-depolarization in a graded manner to suppress the bAP-induced Ca^2+^ transients in the particular dendritic branch.

Finally, we examined how the clustering of GABA receptors at the branch point affected the inhibition of Ca^2+^ transients (Fig. 4C). We found that Ca^2+^ transients were similarly inhibited (Fig. 4Da) even when GABA was uncaged at a branch point 20 µm from the imaging site (Fig. 4Db). To test the effect of clustering of GABA receptor at the branch point, we reduced the laser power to 50% based on the finding that1pIPSC amplitudes at the branch point were 2 times larger than in other dendritic regions (Fig. 1F and 2B), and that the GABA-induced currents were linearly related to the laser power (Fig. 3G). At 50% laser power the inhibition was absent (Fig. 4Dc), indicating that the clustering of GABA receptors at the branch point potentiated the inhibition of the Ca^2+^ transients. The branch point inhibition, however, was absent when the imaging site (IS) was 30–40 µm away from the branch point (Fig. 4Dd). Thus, the higher density of GABA receptors at the branch point contributed to the inhibition of Ca^2+^ transients farther into the oblique dendrites without blocking propagation of the bAP.

## Discussion

We have characterized the distribution and function of GABA_A_ receptors in the dendrites of CA1 pyramidal neurons using 2P and 1P uncaging of caged-GABA compounds. We found that GABA_A_ receptors are diffusely distributed in the dendrites— unlike glutamate receptors [1]— in accordance with previous 1P uncaging experiments [11] and electron microscopic (EM) studies that showed no GABA_A_ receptor clusters at postsynaptic densities and abundant extrasynaptic GABA receptors [14].

We found that GABA-mediated current amplitudes at branch points on the major dendritic trunks were two times larger than those elsewhere (Fig. 1, 2). Since a light microscopy has not an enough spatial resolution to estimate the absolute density of receptors per membrane area at the branch point, our findings should be assessed with EM in the future [14], [15]. The clustered GABA receptors caused larger inhibitory effects on the dendritic Ca^2+^ signaling (Fig. 4B). We speculate that the clustering may also explain the larger inhibitory effect on membrane potential and possibly contribute to the formation of an AND-NOT gate within the dendrites [16]. The functional role of the branch-point inhibition must also depend on the types and firing patterns of interneurons innervating the branch points [17]; their precise role remains to be clarified.

We also found that GABA uncaging at oblique dendrites inhibited the Ca^2+^ transients evoked by a single bAP in a spatiotemporally confined and graded manner. Consistently, it has been reported that a single bAP is not sufficient to evoke regenerative Ca^2+^ spikes in the dendrites [18], [19]. The spatially confined inhibition (<20 µm) is at variance with the greater spread of GABA-mediated IPSPs in the resting state of the dendrites [20]. The spatially confined inhibition is explained by a reduction of the electrical length constant by activation of ion channels, such as voltage-gated Ca^2+^ channels, during the after-depolarization caused by bAP. Thus, GABA-mediated inhibition in the dendrites can be spatially confined within a period shortly after bAP, unlike the inhibition in the resting state [20].

Since bAP-induced Ca^2+^ transients are implicated in spike-timing dependent plasticity (STDP) [19], [21], the spatiotemporally confined GABAergic inhibition may also play a key role in regulation of synaptic plasticity. GABA uncaging is a powerful tool to further clarify dendritic integration and synaptic plasticity.

## Materials and Methods

### Ethics Statement

All animal experiments were performed in accordance with the regulations of the Graduate School of Medicine, the University of Tokyo, and approved by the Animal Experiment Committee (The approval number: 1718T134).

### Electrophysiology

Hippocampal slices with a thickness of 350 µm were obtained from 16–20 day old Sprague-Dawley rats as previously described [1]. The extracellular solution contained 125 mM NaCl, 2.5 mM KCl, 2 mM CaCl_2_, 1 mM MgCl_2_, 1.25 mM NaH_2_PO_4_, 26 mM NaHCO_3_ and 20 mM glucose. This bathing solution also contained 200 µM Trolox (Aldrich, USA). For mapping of GABA currents, voltage-gated sodium-channels and α-amino-3-hydroxy-5-methyl-4-isoxazolepropionic acid (AMPA) receptors were blocked by 1 µM tetrodotoxin (Nacalai Tesque, Japan) and 10 µM CNQX (Tocris, UK). The intracellular solution contained 140 mM CsCl, 2 mM NaCl, 2 mM MgATP, 0.5 mM NaGTP, 2 mM D-ascorbic acid, 10 mM Cs-HEPES, 10 mM EGTA and 0.05–0.10 mM Alexa-594, adjusted to pH 7.3 with CsOH. The cells were held at −70 mV in voltage clamp mode to measure IPSCs. The series resistance was typically <20 MΩ.

We used the first to third branches of the apical dendritic trunk for uncaging and imaging experiments. For the effects of GABA-uncaging on bAP-induced Ca^2+^ transients, the whole-cell patch-pipette solution contained 138 mM K-gluconate, 4 mM MgCl_2_, 10 mM disodium phosphocreatine, 4 mM Na-ATP, 0.3 mM Na-GTP, and 10 mM K-HEPES, 0.1mM OGB-1 and 0.05–0.10 mM Alexa-594 at pH 7.2. Action potentials were evoked by current injection (1 nA, 1 ms). Membrane potentials were kept at about −65 mV in current clamp mode. Data were low-pass filtered at 2 kHz, sampled at 5–10 kHz and recorded using FV1000-MPE software (Olympus, Japan). All physiological experiments were performed at room temperature (23–25°C).

### Two-photon-excitation imaging and uncaging of caged compounds

2P imaging of CA1 hippocampal neurons and 2P uncaging of caged compounds were performed with an upright microscope (BX61WI; Olympus, Japan) equipped with a water immersion objective lens (LUMPlanFL/IR 60×, numerical aperture 0.9) and FluoView FV1000-MPE software (Olympus). The FV1000-MPE was modified to control a diode laser with a wavelength of 473 nm (Olympus) and two mode-locked lasers with wavelengths of 800 nm and 860 nm (Tsunami and MaiTai DeepSee; Spectra Physics, USA). The imaging beam (860 nm) was introduced into one scan head, while the uncaging beams (800 nm and 473 nm) were combined on the same light-path using a dichroic mirror before entering the second scan head. The intensity of each laser was controlled independently by acousto-optical modulators. Caged-GABA compounds BCMACM-GABA (5–6 mM) [10] and RuBi-GABA (0.1–0.2 mM) [11] (Tocris, UK) were applied locally from a glass pipette positioned close to the selected dendrite. The actual concentrations of compounds at the dendrites were estimated to be about half of the values in the pipettes. 2P uncaging of BCMACM-GABA was performed with the 800 nm laser at 8–10 mW for 1–2 ms. 1P uncaging of RuBi-GABA was performed with the 473 nm laser at 0.5–0.8 mW for 2–4 ms. The reversal potential of the GABA currents induced by GABA uncaging at the soma was about −65 mV [11]. Mapping of GABA-mediated currents was performed as described previously for glutamate uncaging [1]. Two-dimensional (2D) maps of the current amplitudes were obtained by uncaging at each pixel, separated by 0.9–1.6 µm (1P) or 2.7 µm (2P) within a region of interest in a pseudorandom fashion [1]. Three-dimensional (3D) maps were constructed with the maximal intensity projection of 2D maps obtained at three z-axis planes separated by 1.5 µm or 5 µm.

Fluorescence emission was acquired at 468–552 nm (green channel) and 590–650 nm (red channel) for OGB-1 and Alexa Fluor 594, respectively. Changes in cytosolic Ca^2+^ concentrations were estimated with Δ*F*/*F*, where *F* is the average fluorescence intensity of OGB-1 before bAP, and Δ*F* is the difference of fluorescence intensity from *F*. Signals were averaged over 4–12 traces. The averaged traces were fitted with an exponential function, and amplitudes of the Ca^2+^ transients were obtained at 25 ms after GABA uncaging. We used MATLAB (Mathworks, USA) and ImageJ (NIH, USA) for data analysis.

### Statistical tests

Statistical analyses were performed as indicated in the text and figures, using two-tailed *t*-test or paired *t*-test.
